# The Role of Income Volatility and Perceived Locus of Control in Financial Planning Decisions

**DOI:** 10.3389/fpsyg.2021.638043

**Published:** 2021-05-31

**Authors:** Johanna Peetz, Jennifer Robson, Silas Xuereb

**Affiliations:** ^1^Department of Psychology, Carleton University, Ottawa, ON, Canada; ^2^Department of Political Management, Carleton University, Ottawa, ON, Canada

**Keywords:** income volatility, saving, financial planning, financial decisions, locus of control

## Abstract

Two studies examine whether income volatility might lead to greater personal financial insecurity and might create a decision environment that discourages planning ahead on personal finances. In Study 1 (*N* = 982), participants who reported more month-to-month variability in their actual income were less likely to have planned for financial contingencies. A lower internal locus of control partially mediated the link between volatility and financial planning decisions in Study 1, and lower internal locus of economic control predicted financial planning decisions independently of volatility. In Study 2 (*N* = 149), participants who were randomly assigned to receive volatile (vs. stable) payments in a simulated work environment were less likely to save their compensation for this work. Again, lower internal locus of economic control predicted financial planning decisions independently of volatility. This is the first study to demonstrate a causal link between income volatility and financial decisions, specifically a heightened tendency to make short-term financial decisions. Both studies also underlined the importance of internal locus of control for financial planning decisions.

## Introduction

Traditional salaried workers and those with predictable working hours can expect a weekly paycheck that is similar from week to week (or month to month). However, self-employed and gig workers might have earnings that are more irregular and experience shifts in their income which are larger and more frequent compared to workers in standard employment. Across advanced economies, approximately one in six workers is self-employed and one in eight is on a temporary contract (OECD, 2018). Gig work may take a wide range of forms, including independent contractors, on-call workers, temporary help agency workers and sub-contracts to other firms (Bureau of Labor Statistics, 2018). Our research focuses on the role this income volatility plays in psychological outcomes (perceived locus of control) and in financial decisions individuals may make (e.g., saving decisions). We propose that income volatility and perceived control over that volatility may each affect financial planning decisions.

## Income Volatility

### Definition and Prevalence

The available research suggests that monthly income volatility is nearly universal when it is defined as even a small shift (5%) above or below usual monthly income levels. In a study of account activity in a sample of 100,000 clients, researchers at JP Morgan Chase found that 89% of individuals experienced income volatility in a 27-month period when volatility is defined as a 5% or greater change in monthly income (Farrell and Greig, 2015). When income volatility was defined more stringently (as a swing of 30% relative to the previous monthly income), 41% of the sampled client accounts experienced this volatility. This finding is broadly consistent with American studies using survey data to measure self-reported monthly income, which find that half of working-age adults have at least 1 month per year of significant income volatility (Bania and Leete, 2009; Maag et al., 2017). Other studies ask respondents to characterize the degree to which their income varies from month to month. Results of these efforts to measure the prevalence of income volatility range between 12 and 18% in Canada (TD Bank Group, 2017) and 10 and 22% in the United States (annual Survey of Household Economics and Decision-making (SHED) Board of Governors, 2017).

Research on month-to-month income volatility has suggested that while income volatility is present at all levels of income (Farrell and Greig, 2015, 2016; Hannagan and Morduch, 2015; Murdoch and Schneider, 2017), it is particularly pronounced among lower income households (Bania and Leete, 2009; Hannagan and Morduch, 2015; Farrell and Greig, 2016; Maag et al., 2017; Murdoch and Schneider, 2017; TD Bank Group, 2017), among younger people (Farrell and Greig, 2016), African-American respondents (Bania and Leete, 2009), and those with lower levels of education (Bania and Leete, 2009). Some unpredictability in ongoing income flows appears to be the reality for a significant share of adults and this share may be increasing (Maag et al., 2017).

### Financial Outcomes

There are several reasons to believe that having a volatile income might lead to greater personal financial insecurity and might create a decision environment that discourages planning ahead on personal finances. Standard models of rational consumer behavior used in economics suggest that persons with volatile incomes should be motivated to save *more* as a way to smooth their consumption and prepare for unexpected costs (Friedman, 1957; Modigliani, 1986; Carroll and Samwick, 1997). However, those experiencing income volatility are less likely to report saving (Fisher, 2010; Pew Charitable Trusts, 2017; TD Bank Group, 2017), to have shorter savings horizons and to have lower motivation to save for retirement (Fisher, 2010). Income volatility has also been associated with other detrimental financial behaviors such as missing bill payments (Farrell and Greig, 2016; TD Bank Group, 2017) and greater risk of mortgage delinquency, even independent of overall income (Diaz-Serrano, 2005). More generally, income volatility seems to be associated with lower self-reported financial well-being and greater financial strain (Pew Charitable Trusts, 2017; TD Bank Group, 2017) and to financial impatience (West et al., 2020). In sum, there is plenty of evidence that people who experience shifts in income also have more negative financial outcomes.

However, the vast majority of this existing work linking income volatility and financial outcomes is purely correlational, so it is possible that those who tend to make worse financial decisions are drawn to volatile income work. We are aware of only one study that has examined the effect of income volatility experimentally (West et al., 2020): A sample of Kenyan women were randomly assigned to a control group or to receive unexpected and positive cash transfers over the course of 6 weeks. Those in the treatment group demonstrated significantly higher financial impatience at the end of this time period, choosing a smaller, hypothetical cash prize today over a larger hypothetical cash prize in 6 months. Although not yet peer reviewed, this preliminary evidence suggests that unexpected changes in income, whether increases or decreases, might have a causal and negative impact on forward planning in personal finances.

## Psychological Consequences of Income Volatility: Locus of Control

Volatile income profiles might also affect people’s general outlook on life. When experiencing constant shifts in income, the uncertainty associated with these shifts and the unpredictability of their income might lead people to develop a lower sense of internal locus of control over time. An internal locus of control is characterized by the belief that one is in charge of one’s own life outcomes, whereas those who have an external sense of control would see their successes and failures as mainly due to external factors such as luck or fate (Rotter, 1966). A sense of locus of *economic* control (Furnham, 1986) references individual beliefs about economic outcomes (e.g., whether one becomes rich or poor) being due to internal (ability, effort) or external (luck, fate) factors. We propose that both the general internal locus of control (Rotter, 1966) and internal locus of economic control (Furnham, 1986) might be lower among people with high income volatility. The experience of variability in income and accompanying financial uncertainty might, over time, reduce people’s sense of internal locus of control.

There is also reason to expect that a weaker internal locus of control might be linked to poorer financial decisions, in turn. The sense of certainty accompanying a greater sense of internal control over any life outcomes, and economic life outcomes specifically, may enable individuals to forego immediate gratification in favor of longer-term financial goals in consumption and saving (Thaler and Shefrin, 1981; Becker and Mulligan, 1997). Such longer-term orientation may include less discounting of money over time (Frederick et al., 2002), and better financial planning outcomes more generally. Indeed, an internal locus of control has been shown to be strongly associated with higher levels of financial capability (Shephard et al., 2017), greater satisfaction with one’s household financial circumstances (Sumarwan and Hira, 1993), more rational financial decision-making (Plunkett and Buehner, 2007), more purposeful shopping habits in Canadian students (Busseri et al., 1998), and higher rates of saving (Cobb-Clark, 2015; Cobb-Clark et al., 2016). For example, households with one respondent who believes that he or she can generally control their own life outcomes save more overall and as a percentage of their income (Cobb-Clark et al., 2016) and are more likely to save (and save more) for their retirement (Schäfer and Konrad, 2016). Thus, income volatility might lead to worse financial outcomes in part because the unreliable shifts in income reduce people’s belief in their own agency and reduce their sense of internal locus of control.

Of course, there are also alternative explanations for the link between income volatility and financial outcomes. An internal locus of control might only be one of many psychological consequences of experiencing frequent shifts in income and only one of the possible mediators of volatility effects on financial outcomes. There are practical reasons for the link between volatility and reduced saving decisions: A more uncertain future income may increase exclusion from mainstream banking and consumer credit (Murdoch and Schneider, 2017) and lead individuals to pay higher transaction costs with fringe banking providers (Buckland, 2012; Servon, 2017). It might also be that receiving income in less predictable amounts actually changes individuals’ decision tendencies toward more short-term thinking, independent of changes to their locus of control and relative to more stable income patterns. When households face uncertainty in their income, they may be conditioned to prefer short-term planning horizons in general (Barr, 2012; Mullainathan and Shafir, 2013). Financial instability might also create the need for constant focus and attention, increasing overall cognitive load and interfering with cognition to make plans or take advantage of opportunities to alleviate the effects of poverty (Gennetian and Shafir, 2015). In sum, income volatility might affect financial planning decisions for a multitude of reasons, one of which may be a lower sense of internal control over one’s fate. In the present research we examine both a general sense of internal locus of control (the extent to which people see themselves in control of life outcomes) and a sense of economic control (the extent to which people see themselves in control of financial outcomes), specifically.

### Locus of Control as Buffer of Income Volatility

There might also be a third aspect of perceived control relevant to the effects of income volatility: the relationship between volatility and financial planning decisions might be attenuated if the volatility itself is perceived as controllable. A person might perceive the shifts in income itself as more or less controllable, depending on the conditions of their paid work. For example, self-employed workers might choose to seek more or fewer contract opportunities in a given month (controllable), whereas other workers rely on a third-party agency to arrange work opportunities, without control over the shifts they are assigned (uncontrollable). In fact, a more internal locus of control has been associated with greater likelihood of pursuing self-employment (Caliendo et al., 2013). Perception of control over income volatility might moderate the behavioral effects of volatility, with detrimental effects of volatility for financial planning being limited to those who perceive less control over the changes to their earnings, but attenuated for those who perceive some control.

## The Present Research

The central aim of the present research is to examine the effects of income volatility with differences in perceived control on individual financial decision making using both correlational and experimental methods. We conceived and conducted this research in two complementary parts. First, we conduct a correlational study to examine whether experiencing more income volatility in daily life is linked to worse financial planning decisions. In part, we hypothesize, this link may be due to a change in people’s psychological outlook on life such as lower internal locus of control in those with volatile incomes (relative to those with stable incomes). Second, we conduct an experimental study to determine whether volatility has a causal effect on financial planning behavior, and whether a greater sense of control over the shifts in income protects from the adverse effects of income volatility. We propose that those with volatile incomes (relative to those with stable incomes) might demonstrate worse financial decisions only if the volatility is outside their control but not if they perceive control over the shifts in income.

In our correlational study, we test the association between real income volatility, psychological outlook and financial planning in a large community sample (Study 1), measuring four financial planning decisions. In a second study, we experimentally manipulated the income earning experience in a community sample (Study 2). In this study, participants were randomly assigned to work in a manner that simulates an uncontrollable volatile income, a controllable volatile income, or a stable income. We measured participants’ financial decisions via their preference to receive lower pay now or wait for higher payout two weeks later, a proxy measure for willingness to save. Both studies are conducted using North-American samples of adults in highly developed economies with liberal welfare regimes. However, our interest is primarily in the psychological effects of income volatility at the individual level, not on cross-country comparisons of economic or political determinants of volatility.

## Study 1

In an initial test of the correlates of income volatility, a large sample of North-American participants reported their income, the month-to-month volatility of this income, and the degree of perceived control over this volatility. We then assessed perceived locus of control (general and specific to economic outcomes) and four financial planning decisions: keeping a budget, having made retirement plans, having insurance, and using savings when confronted with an unexpected cost. We expected that, in line with previous studies showing poorer financial outcomes for individuals with high income volatility (Diaz-Serrano, 2005; Farrell and Greig, 2015, 2016; Pew Charitable Trusts, 2017; TD Bank Group, 2017), participants’ reported income volatility would be linked to less planful behaviors in their personal finances. We also expected that participants’ reported income volatility would be linked to a weaker internal locus of control, and that this belief would in turn be linked to less planful financial decisions. Finally, we expected that participants’ perceived control over the volatility in their income would moderate the effects of volatility, such that volatility might not be associated with poorer financial planning decisions among those individuals who perceive high control over the volatility.

### Method

#### Participants

We recruited 1,005 American participants through the recruitment platform Mechanical Turk for this online survey (in July 2019). Of these, 23 participants were excluded from analysis for failing an attention check, resulting in a final *N* of 982. Participants’ age ranged from 18 to 76 (*M*_*age*_ = 37.03, SD = 11.45), 46.3% were female, 53.2% were male, and 0.5% classified their gender as other. Education level ranged from high school or less (11.2%), over some college/university without a degree (23.5%) to college/trade degrees (19.7%), undergraduate degrees (32.1%) and graduate degrees (13.6%). Personal annual income ranged from under $20,000 (23.4%) to over $100,000 (8.4%), average income was $40,000-$50,000. Table 1 presents more detailed demographic information.

**TABLE 1:** Demographic characteristics of the samples in percent.

	Study 1 Sample %	Study 2 Sample %	U.S. Population %	Canadian Population %
*Age*				
18-24	8.4	28.2	11.8	11.1
25-44	68.2	52.3	34.3	33.7
45-64	21.3	15.5	33.4	33.5
65+	1.9	4.0	20.5	21.7
*Gender*				
Male	53.2	38.9	48.4	49.4
Female	46.3	59.7	51.6	50.6
Other	0.5	1.3	0.0	0.0
*Education level*				
High school or less	11.2	14.1	39.5	35.2
Some post-secondary	43.2	31.5	28.2	36.3
Bachelor’s degree or higher	45.7	54.4	32.3	28.5
*Household income*				
Under $20,000	23.4	41.9	29.1	32.2
20,000 - $39,999	26.0	17.6	24.6	20.9
40,000 - $59,999	21.3	13.5	17.0	17.6
60,000 - $99,999	20.8	22.3	16.3	19.2
Over $100,000	8.5	4.8	12.9	10.0
*Marital status*				
Married/common-law	45.7	24.2	47.8	47.5
Single	44.1	65.8	33.7	40.3
Separated/divorced/widowed	10.2	10.1	18.5	12.2

*Percentages may not add up to 100% due to rounding. Population percentages according to Statistics Canada (2017, 2021a,b,c), U.S. Census Bureau (2018, 2020, 2021).*

#### Procedure

Full materials are available at https://osf.io/ja5m4/. The study was reviewed and approved by the Carleton University Research Ethics Board. First, participants completed a consent form and reported demographic information. They reported their age, gender, education in 5 categories and personal annual income before taxes in 11 categories (from under $20,000 to $100,000 or more).

##### Income Profile

Next, participants reported on the nature of their income. Participants rated the amount of volatility (“How much does the amount of money you make change from month to month?”) on a scale from 1 (*Amount of income is the same every month*) to 7 (*Amount of income changes a lot from month to month*). They also rated the amount of control they perceived over this volatility (“To what degree do you feel you can control how much money you make in a month?”) on a scale from 1 (*I have no control at all*) to 5 (*I have all the control*)^1^.

##### Locus of Control

General locus of control was assessed with 14 statements assessing belief in internal locus of control (e.g., “What happens to me is my own doing”). These items were taken from the Internal-External Locus of control scale (Rotter, 1966). Responses were measured on a scale from *Strongly disagree* (1) to *Strongly agree* (7). Six of the items were reverse coded, and all items were then averaged into an index of internal locus of control belief (α = 0.78).

Locus of economic control was assessed with 4 statements assessing belief in internal locus of economic control (e.g., “It is chiefly a matter of fate whether I become rich or poor.” reverse coded). These items were taken from the Economic Locus of Control Scale (Furnham, 1986). Responses were measured on a scale from *Strongly disagree* (1) to *Strongly agree* (7). Two of the items were reverse coded, and all items were then averaged into an index of internal locus of economic control belief (α = 0.67). The two scales, for each general and locus of economic control, correlated at *r* = 0.51, *p* < 0.001.

##### Financial Decisions

Participants completed a number of questions about financial behaviors (Robson and Splinter, 2015). Of these, we examined four items that assessed planning decisions, specifically whether participants had a household budget (64.3% said yes), whether they have a retirement plan of any sort (66.6% said yes or were already retired), whether they hold any insurance policies (75.7% said yes), and, in response to how they would cover a large unexpected cost equivalent to two weeks of take-home pay, whether they said they would use savings rather than borrow money, sell possessions, or that they couldn’t cover that cost (44.3% said they would use savings). We aggregated these four financial decisions (budgeting, long-term planning, self-insuring against risk, and planning for unexpected costs) on a 0-4 scale where 0 = *none of these planning decisions are present* and 4 = *all these planning decisions are present* (*M* = 2.46, *SD* = 1.23). Responses were normally distributed.

### Results

#### Initial Analyses

Income volatility was significantly and negatively correlated with age (*r* = −0.12, *p* < 0.001), education (*r* = −0.09, *p* = 0.003), amount of income (*r* = −0.27, *p* < 0.001), and was significantly higher for female than male participants [*t*(972) = 2.49, *p* = 0.013]. Thus, in all analyses below we control for these demographic variables as covariates, to ensure that any association between income volatility and financial decisions is not capturing variation in these demographic variables.

#### Income Volatility and Financial Planning Decisions

In a multiple regression analysis, we entered income volatility and demographic control variables as predictors and the financial planning decision index as our dependent variable^2^. Table 2 presents our results. Individuals reporting more income volatility also reported fewer financial planning decisions, *B* = −0.10, *SE* = 0.02, β = −0.15, *p* < 0.001, independent of the person’s age, gender, education, and the amount of their personal income^3^.

**TABLE 2:** Regression estimates for the link of income volatility with the combined financial planning decisions, internal Locus of Control, internal Locus of Economic Control, controlling demographic variables.

	Financial Planning Decisions		Internal Locus of Control		Internal Locus of Economic Control	
			
	*B (SE), beta*	*p*	*B (SE), beta*	*p*	*B (SE), beta*	*p*
Volatility	−0.10 (0.02), −0.15	<0.001	−0.05 (0.01), −0.14	<0.001	−0.08 (0.02), −0.15	<0.001
Age	0.02 (0.003), 0.16	<0.001	0.01(0.002), 0.14	<0.001	0.01(0.003), 0.11	0.001
Female	0.10 (0.07), 0.04	0.165	−0.08 (0.05), −0.05	0.093	0.02 (0.07), 0.01	0.819
Education	0.14 (0.03), 0.14	<0.001	−0.07 (0.02), −0.12	<0.001	−0.07 (0.03), −0.08	0.013
Income	0.12 (0.01), 0.27	<0.001	0.06 (0.01), 0.22	<0.001	0.05 (0.01), 0.12	<0.001

#### Income Volatility and Perceived Locus of Control

Next, we examined the link between income volatility and each locus of control in multiple regressions (see Table 2). Greater income volatility was associated with lower internal locus of control, *B* = −0.05, *SE* = 0.01, β = −0.14, *p* < 0.001, and also lower internal locus of economic control, *B* = −0.08, *SE* = 0.02, β = −0.15, *p* < 0.001, after controlling for the influence of age, gender, education, and amount of income.

**FIGURE 1:**
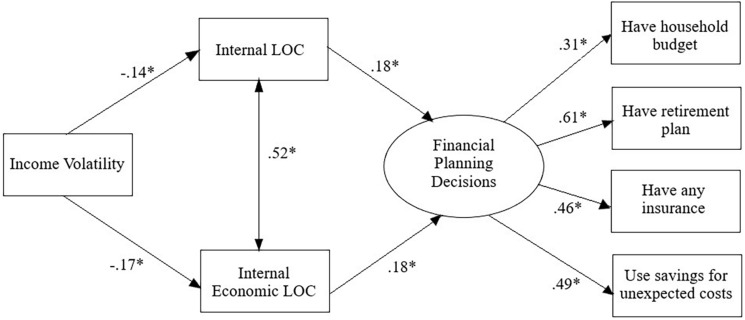
Model presenting standardized coefficients. For ease of presentation, the covariates (age, gender, income, education) and error variances are not depicted, although they were included in the model. ^∗^*p* < 0.01. LOC = Locus of Control.

We then tested the indirect links of volatility on financial planning decisions via perceived locus of control. Using structural equation modeling (AMOS v.27), we tested a path model where income volatility was linked to locus of control and locus of economic control, which were in turn linked to a latent variable denoting financial planning decisions (Figure 1). We controlled for the influence of age, gender, income, and education on all variables. Results of this SEM analysis were consistent with the findings from the simple correlations and multiple regressions. Higher income volatility was significantly linked to lower internal locus of control, β = −0.14, *p* < 0.01, and lower internal locus of economic control, β = −0.17, *p* < 0.01. Both were independently and positively linked to the latent factor denoting financial planning decisions, βs = 0.18, *p* < 0.01. A considerable portion of the variance in the latent factor was explained by the model (44%). Model fit was acceptable, RMSEA = 0.07, CFI = 0.91^4^.

#### Income Volatility and Perceived Control Over the Volatility

Finally, we examined whether the control workers feel over the changes in their income might attenuate the negative link between volatility and financial planning. In multiple regression, we entered volatility, perceived control over volatility, and their interaction term as predictors, and the financial planning index as dependent variable. Predictors were centered before analysis. We again entered demographic variables (age, gender, education, and income amount) as covariates. Results are presented in Table 3. The volatility by control interaction term was not significant, *B* = 0.01, *SE* = 0.02, β = 0.02, *p* = 0.513. However, more volatility was linked to fewer financial planning decisions, *B* = −0.11, *SE* = 0.02, β = −0.17, *p* < 0.001, and more perceived control over the income volatility was also – independently from volatility - linked to more financial planning decisions, *B* = 0.18, *SE* = 0.04, β = 0.13, *p* < 0.001.

**TABLE 3:** Regression estimates for link of income volatility and control over volatility with the combined financial planning decisions, controlling demographic variables.

	Financial Planning Decisions		
	
	*B (SE)*	*beta*	*p*
Income Volatility	−0.11 (0.02)	−0.17	<0.001
Control over Volatility	0.18 (0.04)	0.13	<0.001
Volatility × Control	0.01 (0.02)	0.02	0.513
Age	0.02 (0.003)	0.17	<0.001
Female	0.13 (0.07)	0.05	0.083
Education	0.13 (0.03)	0.14	<0.001
Income	0.11 (0.01)	0.25	<0.001

### Discussion

Our correlational study showed a negative link of income volatility with financial planning decisions, in line with previous studies showing poorer financial outcomes for individuals with high income volatility (Diaz-Serrano, 2005; Farrell and Greig, 2015, 2016; Pew Charitable Trusts, 2017; TD Bank Group, 2017). This link was at least partially explained by a lower internal locus of control and lower internal locus of economic control– a weaker sense of being in charge of one’s own (economic) fate. This is the first study to show that a financial decision environment (the extent to which income shifts from month-to-month) is linked to the psychological perception of the world, specifically locus of control. Finally, contrary to our expectation, participants’ perceived control over the volatility in their income did not moderate the effects of volatility. The association between higher income volatility and less planful financial decisions was not attenuated when people perceived control over the volatility. Perceived control over any income volatility (whether low or high) was linked to more planful financial decisions but did not, in the interaction, reduce the effects of higher volatility alone. In sum, this study underscored the importance of perceived locus of control – in general, with regard to finances, and with regard to one’s income shifts – for financial planning decisions. Next, we examine whether this relationship is causal using an experimental design.

## Study 2

We propose that the experience of month-to-month shifts in income volatility creates a lower internal sense of control and discourages financial planning decisions. Study 1 assessed these variables correlationally and did not establish a causal link. It is possible that individuals who tend to have a low internal sense of control or who tend to not engage in financial planning are also more likely to take up the type of work that pays irregularly. In Study 2 we examined the effect of income volatility experimentally. Participants were randomly assigned to complete simulated work with volatile payouts or stable payouts. We further manipulated the control participants had over picking tasks (i.e., they could control the shifts in payment somewhat or not at all). We then assessed a specific, meaningful, planning decision: whether participants decided to delay receiving their actual pay for a higher amount or whether they chose immediate payout for a lower amount.

### Method

#### Participants

We recruited 152 community participants in Canada. The sample size was determined *a priori*. Of these, 2 participants did not finish the study and 1 participant was unable to read or understand the questions. These participants were excluded from analysis, resulting in a final *N* of 149. Power sensitivity analyses (G^∗^power) suggest our final sample had 80% power to detect medium effect sizes (e.g., *f* = 0.25, *d* = 0.5) for one-way ANOVAs comparing three conditions (assuming a significance level of *p* < 0.05) and 99% power to detect large effects (e.g., *f* = 0.38, *d* = 0.76). Participants were paid $15 as compensation for their time.

Participants’ ages ranged from 18 to 76 (*M*_*age*_ = 33.85, *SD* = 14.06), 59.7% were female, 38.9% were male, and 1.3% participants selected “other” for gender, 75.9% were single, separated, divorced or widowed, 24.2% were married or living common law. Education level ranged from high school or less (14.1%), over some college/university without a degree (17.4%) to college/trade degrees (14.1%), undergraduate degrees (28.2%) and graduate degrees (26.2%). Personal annual income ranged from under $20,000 (41.9%) to over $100,000 (4.4%), average income was $30,000-$40,000. Table 1 presents more detailed demographic information.

#### Procedure

The study was reviewed and approved by the Carleton University Research Ethics Board. Participants were recruited via posters and flyers in the local community, and online posts in local volunteer forums. They signed up via a website or by emailing the researchers. The study was conducted in public spaces between August 2019 and January 2020. Participants completed the study one-on-one with a research assistant. They answered all questions and completed the simulated work on mobile devices provided by the research assistant.

##### Initial Measures

Participants completed a consent form and a range of demographic and financial behavior measures, as in Study 1. Full materials are available in online supplements at https://osf.io/ja5m4/. Most importantly, they completed the same measures of locus of control (Rotter, 1966, α = 0.59) and locus of economic control (Furnham, 1986, α = 0.54) as in Study 1.

##### Simulated Work

Next, participants took part in a task that simulated different types of work and income. For about 30 min, participants completed a variety of tasks (e.g., mental and visual puzzles) for which they received “points” as payment (details of tasks^5^). They were informed that the points would later be exchanged for real money, so their earnings from the simulated task would actually be paid out to them. In the uncontrollable volatile condition (*n* = 50), tasks paid out different amounts of points and participants received very different payouts (in points) across three “work periods” (i.e., high volatility). Tasks were assigned to these participants without their input (i.e., low control). In the controllable volatile condition (*n* = 50), tasks paid out different amounts of points and participants received very different payout (in points) across three “work periods” (i.e., high volatility) but participants could choose whether to complete or refuse tasks based on the nature of the task and the points they would earn (i.e., high control). In the stable income condition (*n* = 49), participants received the same payout across three “work periods,” and in each period, participants were simply given all tasks and told to work through them at their own pace. To eliminate the potential for an effect on savings decisions from the amount of the final payment itself, all participants received the same dollar amount in compensation regardless of the work simulation they were randomly assigned to.

##### Manipulation checks

As a manipulation check, participants reported perceived volatility of the simulated income on two items [e.g., “How much did the payout (i.e., points/“income”) change between work periods?,” *r* = 0.34] on 5-point scales, and reported perceived control over the simulated work on three items (e.g., “How much control did you feel you had over the number of points earned?,” α = 0.66) on 5-point scales. As additional measures of task experience, participants also reported enjoyment (“How much did you enjoy the income game?”) and stress (“How stressed did you feel during the income game?”) on single items on scales from 1 (*Not at all*) to 5 (*Very much*).

##### Post-task Measures

Directly after the simulated work, participants completed another measure of locus of control (14 “version B” statements from the original Rotter, 1966 scale, α = 0.52) and the other 4 items of the locus of economic control (Furnham, 1986, α = 0.66).

##### Financial Saving Decision

After finishing the simulated work, participants were paid for their work (i.e., in addition to the money they received as compensation for their time). Everyone received $15 regardless of the points accumulated in the session. After being informed of their payment, participants were given a choice: They could choose to take the $15 now, or to “save” their earnings by waiting two weeks and receiving $17 ($15 earnings plus $2 in “interest”). A decision to delay payment and engage in saving would be indicative of more planful behavior. In each case, participants were paid the amount in gift cards to either Amazon or Walmart and the gift card was ordered immediately, with the participant putting in their own email address and the research assistant putting in the amount and the date on which the gift card should be sent. This was done to avoid conflating trust in the research assistant (e.g., thinking he/she might not remember to send it later) and willingness to wait for higher payouts. Similarly, we chose online gift cards rather than cash to avoid conflating effort (e.g., having to come back in person to collect cash) and willingness to wait for higher payouts. The amount and delay was based on a pilot sample of 30 participants who were recruited from a student participant pool. A delay of only one week led to a ceiling effect of saving: only 10% chose immediate payout, we therefore doubled the delay for the actual study.

### Results

#### Initial Checks

There were no differences across the experimental groups for demographic variables such as age, *F*(2,146) = 0.68, *p* = 0.509, η^2^ = 0.009, gender, *X*^2^ (*df* = 1, *N* = 149) = 2.15, *p* = 0.342, education, *F*(2,146) = 0.05, *p* = 0.951, η^2^ = 0.001, and their actual annual income, *F*(2,145) = 0.53, *p* = 0.591, η^2^ = 0.007, or volatility of their actual income outside of the experimental setting, *F*(2,145) = 0.17, *p* = 0.843, η^2^ = 0.002, suggesting that random assignment to conditions was successful. Consequently, we do not control for these demographic variables when examining the effect of the experimental manipulation.

#### Manipulation Checks

We found that as intended, condition affected perceived controllability of the simulated work, *F*(2,144) = 4.99, *p* = 0.008, η^2^ = 0.065, and perceived volatility of the simulated income, *F*(2,144) = 11.49, *p* < 0.001, η^2^ = 0.139. See Table 4 for Means. The simulated work was seen as more volatile in the two volatile conditions than in the stable condition. The simulated work was perceived as less controllable in the uncontrollable volatile condition than in the controllable volatile condition. There were no differences in terms of enjoyment, *F*(2,144) = 0.57, *p* = 0.568, η^2^ = 0.008, or stress, *F*(2,144) = 0.71, *p* = 0.493, η^2^ = 0.010, during the simulated work indicating that the simulated work differed only in the two relevant aspects, income volatility and perceived control over this income.

**TABLE 4:** Observed means by experimental condition.

	Uncontrollable volatile income condition	Controllable volatile income condition	Stable income condition
Volatility of income for the simulated work	3.46_a_ (0.74)	3.44_a_ (0.64)	2.79_b_ (0.92)
Perceived control over income	2.61_a_ (0.92)	3.22_*b*_ (0.99)	2.88_ab_ (0.96)
Enjoyment of simulated work	3.82_a_ (1.06)	3.65_a_ (1.14)	3.86_a_ (0.91)
Stress during simulated work	2.98_a_ (1.29)	2.77_a_ (1.31)	2.67_a_ (1.33)
Pre-simulated work LOC	4.14_a_ (0.56)	4.14_a_ (0.58)	3.97_a_ (0.58)
Post-simulated work LOC	3.94_a_ (0.60)	3.95_a_ (0.57)	3.90_a_ (0.52)
Pre-simulated work economic LOC	5.11_a_ (0.98)	5.23_a_ (0.90)	5.01_a_ (0.89)
Post-simulated work economic LOC	4.90_a_ (1.06)	4.57_a_ (1.16)	4.70_a_ (1.01)

*Standard Deviation in parentheses. Subscripts identify significant contrasts: means that share the same subscript within rows are not statistically different, means with different subscripts are significantly different at *p* < 0.05. All variables measured on 5-point scales ranging from 1 to 5. LOC = Locus of Control.*

#### Financial Saving Decision

Next, we examined participants’ willingness to delay payment and “save” after the simulated work to receive a higher dollar amount. Participants in the stable condition were more likely to choose the delayed payout (84%) than participants in the uncontrollable volatile condition (68%) or controllable volatile condition (68%). The difference between the two volatile groups and the stable group was significant, *X*^2^ (*df* = 1, *N* = 149) = 4.11, *p* = 0.043. The number of participants who chose delayed payout was identical in the two volatile conditions, *X*^2^ (*df* = 1, *N* = 100) = 0, *p* = 1. In sum, the experimental manipulation of simulated volatile vs. stable income had a significant effect on participants’ real financial choice, suggesting a causal effect of income volatility on planning decisions.

#### Locus of Control

We conducted a 2 (time: pre, post) by 3 (condition) mixed ANOVA to examine whether any change in participant locus of control, measured before and after the simulated work, differed by condition (Table 4). The main effect of time was significant, *F*(1,146) = 11.78, *p* < 0.001, η^2^ = 0.075, with participants scoring lower in the version of the locus of economic control scale assessed after the simulated work than in the version assessed as baseline. The main effect of condition was not significant, *F*(2,146) = 0.74, *p* = 0.478, η^2^ = 0.010. The interaction term was not significant, *F*(2,146) = 0.92, *p* = 0.400, η^2^ = 0.012.

We also examined whether locus of control or change in locus of control predicted the saving decision. In a logistic regression, we entered the condition as a dummy variable (*volatile conditions* = *1, stable condition* = *0*), the post-work version of locus of control, and the difference score between the pre- and post-assessments to indicate change in locus of control as predictor variables, and entered saving decision as outcome variable. Condition had a significant effect, *B* = −0.96, *SE* = 0.45, *B(Exp*) = 0.38, *p* = 0.034, locus of control had a marginally significant effect, *B* = 0.72, *SE* = 0.39, *B(Exp)* = 2.05, *p* = 0.065, and the difference score had no significant effect, *B* = −0.25, *SE* = 0.40, *B(Exp)* = 0.78, *p* = 0.530.

#### Locus of Economic Control

We conducted a 2 (time: pre, post) by 3 (condition) mixed ANOVA to examine whether any change in participant locus of economic control, measured before and after the simulated work, differed by condition (Table 4). The main effect of time was significant, *F*(1,145) = 31.07, *p* < 0.001, η^2^ = 0.176, with participants scoring lower in the version of the locus of economic control scale assessed after the simulated work than in the version assessed as baseline. The main effect of condition was not significant, *F*(2,145) = 0.34, *p* = 0.715, η^2^ = 0.005. The interaction term was significant, *F*(2,145) = 3.85, *p* = 0.023, η^2^ = 0.050, with a proportionally greater drop in locus of economic control in the controllable volatile condition than the other two conditions. This effect was unexpected, as this condition was actually intended and perceived, as indicated in the manipulation checks reported earlier, to give *more* control over the shifts in “income” during the simulated work. It is possible that experiencing greater control as part of the simulated work in the experiment highlighted the lack of perceived control in their actual economic situation, outside of the experimental setting, for these participants (i.e., a contrast effect).

We also examined whether locus of economic control or change in locus of economic control predicted the saving decision. In a logistic regression, we entered the condition as a dummy variable (*volatile conditions* = *1, stable condition* = *0*), the post-work version of locus of economic control, and the difference score between the pre- and post-assessments to indicate change in locus of economic control as predictor variables, and entered saving decision as outcome variable. Condition had a significant effect, *B* = −0.98, *SE* = 0.45, *B(Exp*) = 0.38, *p* = 0.031, locus of economic control had a significant effect, *B* = 0.42, *SE* = 0.21, *B(Exp)* = 1.53, *p* = 0.045, and the difference score had no significant effect, *B* = −0.39, *SE* = 0.27, *B(Exp)* = 0.68, *p* = 0.144. Thus, just as in Study 1, a more internal locus of economic control was linked to more long-term planning– the decision to save the “income” for a higher later payout, even after accounting for the condition effect^6^.

### Discussion

In an experimental test of the causal effect of income volatility, we found after only a 30-min task of simulated volatile income participants were less likely to “save” by postponing an immediate payout for a higher, later, payout than participants who completed similar tasks with stable payouts. Our results in Study 2 are consistent with those of Study 1 which showed this effect correlationally. It is also consistent with previous studies showing that volatility in individuals’ real incomes can lead to shortened planning horizons (Barr, 2012; Mullainathan and Shafir, 2013) and reduced personal savings (Fisher, 2010; Pew Charitable Trusts, 2017; TD Bank Group, 2017). However, this is the first study to show the effect of income volatility in an experimental setting where it is possible to attribute causality.

Contrary to our expectations, we found no difference in saving decisions between participants who were given some control over the volatility in rewards for the simulated work and those who had no control over the volatility. Although Study 1 had found a direct effect of perceived control over the shifts in income on financial planning decisions, the sense of control simulated in our experiment did not have the same effect. The sense of control simulation might not have been strong enough to meaningfully affect the decision (while significant, the average sense of control over the simulated income differed by less than 1 point on a 5-point scale between the two conditions).

Finally, the simulated work also did not shift participants’ locus of economic control as expected – perhaps because locus of control is a belief that is relatively stable and not easily changed within a single experimental session, or because the experiment induced a contrast rather than an assimilation effect. Participants’ sense of internal locus of economic control did, however, once again predict the saving decision, attesting to its importance in financial planning decisions.

## General Discussion

The results of two studies suggest and then confirm experimentally that income volatility has detrimental effects on individuals’ financial planning decisions. Participants who reported more month-to-month variability in their actual income were less likely to have planned for financial contingencies (Study 1) and participants who were randomly assigned to receive volatile (vs. stable) payments in a simulated work environment were less likely to save their compensation for this work for a higher delayed payout (Study 2). To our knowledge, Study 2 is the first study to demonstrate a causal link between income volatility and financial decisions, specifically a heightened tendency to make short-term financial decisions. Both studies also underlined the importance of one psychological consequence of income volatility: the degree to which people perceived internal control of general life outcomes and economic outcomes. Both general locus of control and economic locus of control predicted participants’ saving decisions independent of the simulated work condition. We also investigated a third type of perceived control: control over the changes in income itself. However, we did not find evidence that a sense of control over actual changes in income (Study 1) or giving participants control over the changes in simulated income (Study 2) moderated the relationship between volatility and financial decision-making.

### Theoretical Contributions

These studies are the first to examine the relationship between income volatility and psychological outcomes such as perceived locus of control. The link between income volatility and general locus of control as well as locus of economic control (Study 1) underlines that life circumstances can shape our beliefs about the world. Other life circumstances have been shown to affect locus of control. For example, coronary patients who returned to work reported a more internal locus of control (Bergvik et al., 2012) and workers in jobs that allow for more autonomy reported a more internal locus of control (Wu et al., 2015). The present research adds to the locus of control literature by showing another environmental factor influencing these beliefs: the consistency in which people receive payment for their work (independent of the amount of pay). This work also ties into classic research on operant conditioning (see Staddon and Cerutti, 2003 for a review), showing the detriments of inconsistent reward schedules for financial decisions.

The present research also contributes to the literature on financial decisions, showing a significant impact from volatility in earnings after just 30 min of simulated work on the decision of whether or not to take immediate or delayed and higher compensation. For working-age adults whose income-earning conditions, for example in gig work or other forms of precarious employment, may mean exposure to income volatility for much longer periods of time, its pernicious effects might be much larger outside the laboratory. Future studies might also assess the history of people’s income profiles – having lived with income volatility for longer periods of time might have a measurably larger effect on psychological and financial outcomes.

### Limitations

In Study 1, we used self-reported income volatility on a subjective scale. Administrative and survey data may yield different results in measuring income volatility (Dahl et al., 2011). Self-reported information may overestimate volatility compared to objective measures tracking income shifts via bank deposit history (e.g., Farrell and Greig, 2015), but arguably, self-reported income volatility is more important – *perceived* volatility might matter more psychologically than *actual* changes in income.

In Study 2, locus of control measures had low internal consistency (Cronbach’s alpha between 0.52 and 0.66). The reason for this low internal consistency might be that the established scales (Rotter, 1966; Furnham, 1986) were split into shorter scales to be assessed before and after the simulated work without repeating items. Scales with fewer items tend to have lower reliability (Hattie, 1985). Indeed, when aggregated across all items assessed before and after the work task, alphas are considerably higher (locus of control: α = 0.72, economic locus of control: α = 0.77). Another possibility is that error variance was introduced by participant fatigue due to the length of the study.

### Representativeness of Samples

Both studies relied on convenience samples rather than representative samples of adults in North America (Study 1: United States; Study 2: Canada). Results might not generalize to the population samples were drawn from. For instance, participants were overwhelmingly of working age (>96%) and results therefore do not generalize to retired individuals who might experience less income volatility (e.g., due to pension payout) or who might make very different planning decisions due to their different life situations. In Study 1, which examined the effects of real (rather than simulated, lab-based) income volatility, it is important to note that the percentage of people reporting volatility in their income (13% stated their income varied “a lot” from month to month) was within the range of previous estimates of volatile earners in the population (10-22%, Board of Governors, 2017) and they also reported comparable income amounts (see Table 1 for a comparison of sample and population demographics). However, the sample in Study 1 was more educated and consisted of slightly more men than the general United States population; both these sample-population differences lower the external validity of the results. Education might matter because university education might teach people longer term planning and increase the sense of control they possess. Gender might matter because there might be gender norms for roles in family budgeting, and gender inequalities in earned income. While we controlled for demographic variables in Study 1 to address potential influences of demographic differences, we also conducted exploratory analyses separately by male and female participants and separately by those with and without postsecondary education. Results are reported in the online supplements at https://osf.io/geak8/. Results were robust across these subgroups, suggesting that overrecruiting male and educated participants is unlikely to have had a substantial effect on the reported links between volatility, locus of control and planning decisions. We did find one difference between these subgroups such that women and those with post-secondary education did not show a significant indirect effect of income volatility on planning decisions via economic locus of control (though the indirect effect of general locus of control was significant).

Study 2 focused on testing the causal link between volatility and saving decisions in a lab experiment. The study had good internal validity as shown in manipulation checks, but the simulated work was artificial and the saving decision contrived, lowering external validity along with a biased sample. The sample differed from the general Canadian or American population in several ways, with participants being younger, more educated, more likely to be in the lowest income bracket, and more likely to be female than North-American population averages (Table 1). These characteristics likely occurred because the study was relatively onerous (e.g., requiring physically meeting a research assistant) and did not pay that well ($15 for 1 h time, plus $15 “income” from the simulated work), and was thus likely less appealing to older, wealthier people. It is possible that these sample characteristics increased the effect of the volatility manipulation on saving decisions because younger adults who have spent more time in educational settings rather than the work force might not have as much work experience and are thus more influenced by the simulated work. Younger, lower income participants might also value the simulated “income” more and thus receiving it immediately might be a more tempting option than it would be for someone older who has a higher income outside of the study. Thus, we note that the experimental study only provides initial evidence that temporary experiences of simulated work payout volatility can change one-time saving decisions in this particular sample. However, taken together with the more externally valid Study 1, the similarity of results across these two studies is notable. A study that could examine the causal effect of volatility with greater external validity might manipulate the payout of actual work (rather than a 30 min work period with highly artificial tasks) over a longer time period among a more representative sample of participants.

While the two studies both include North-American samples of adults in highly developed economies with liberal welfare regimes, it is also important to note that the two countries our samples were drawn from differ in several ways, for example in terms of the social safety net available to citizens (e.g., public health insurance in Canada). Because our primary interest is in the psychological effects of income volatility at the individual level, including an experimental design that controlled the exposure to volatility, we do not explore cross-country differences or speculate on the effects of economic or political determinants of income volatility. Future studies may wish to replicate the results with attention to differences in economic climate, welfare regime or other country-level variables.

## Practical Implications

An important share of workers earn their income in so-called gig work. In Canada, the share of workers engaged in on-demand and freelance work has been increasing for the last decade and may increase again in the current recession (Jeon and Ostrovsky, 2020). Added to this are self-employed workers and persons in precarious employment where shifts or wages from one period to the next are uncertain. Furthermore, outside of paid employment, income volatility may also be common among households who rely on income-tested public benefits like temporary employment insurance or residual welfare where amounts can change due to program rules (Robson and Shaban, 2021).

Our present studies confirm previous work (Fisher, 2010; Barr, 2012; Mullainathan and Shafir, 2013; Pew Charitable Trusts, 2017; TD Bank Group, 2017) that income volatility can have detrimental effects on personal financial planning. Volatility, even if it is perceived to be controllable by a worker, may reduce budgeting, self-insurance against risks, and savings for both short-term and long-term needs. This may leave workers and families more financially vulnerable, over and above the direct effect of precarious or uncertain paid work.

These findings may have important practical implications. For practitioners in financial services, some existing products and services (e.g., automated savings or investment plans) may not be appropriate for clients whose incomes rise and fall unpredictably. Can financial products and services be adapted or created to better support the financial well-being of clients who cannot count on the same pay-cheque each month? The results also suggest, for policymakers in government, that we may not be able to expect citizens with volatile incomes to engage in planful financial behaviors to the same extent as those with stable incomes. Policymakers might take steps to reduce income volatility, for example in the regulation of employment markets to ensure workers have greater predictability in their paid hours and working conditions. Policymakers may also need to adjust the design of income support programs and transfers paid to working-age adults and families to provide greater predictability in household incomes. Current programs (such as child benefits and working income credits) that adjust only to year-over year changes in income may not be sufficiently responsive to within-year shocks to income. Similarly, tax incentive programs (e.g., those designed to encourage saving for education and retirement) convey benefits in lump sums, which may reinforce inequality, especially for volatile income earners.

On the psychological side, educators and consumers themselves might find that fostering a sense of internal locus of control can have concrete benefits in promoting longer-term financial planning decisions. In educational and clinical settings, interventions to shift locus of control have been shown to be effective (see Lefcourt, 2014, for a review, also see Gottschalk, 2005). Our work shows that other domains, such as financial cognition, may also benefit from a shift toward internal locus of control.

The global economy has suffered the most acute shock, due to COVID, on record. As economies navigate a pathway to partial and eventual recovery in the wake of the virus, we may find that more work has been converted to on-demand forms of labor where a worker can’t be certain of their take-home pay from one period to the next. We might also see greater uncertainty and a perceived loss of control amidst frequent changes in pandemic-related rules that impact work and earned income. Both of these factors – volatility and locus of control - can have behavioral effects on individuals that we shouldn’t ignore.

## Data Availability Statement

The raw data supporting the conclusions of this article will be made available by the authors, without undue reservation.

## Ethics Statement

The studies involving human participants were reviewed and approved by the Carleton University Research Ethics Board B. The participants provided their informed consent to participate in this study.

## Author Contributions

JP did the conceptualization, performed the methodology, wrote the original draft, reviewed and edited the manuscript, and carried out the formal analysis. JR did the conceptualization, performed the methodology, wrote the original draft, and reviewed and edited the manuscript. SX carried out the project administration, performed the data curation and wrote, reviewed, and edited the manuscript. All authors contributed to the article and approved the submitted version.

## Conflict of Interest

The authors declare that the research was conducted in the absence of any commercial or financial relationships that could be construed as a potential conflict of interest.

## Footnotes

1 Participants were also asked to self-categorize their income profile: “Overall, which of these three income types describes your personal situation best?” and selected one of three options: “My income is stable,” “My income varies and I have no control over how it varies,” or “My income varies and I have control over how it varies”. Participants self-classified their own income as uncontrollable volatile (*n* = 152, 15.5%), controllable volatile (*n* = 363, 37%), or stable (*n* = 467, 47.6%). Analyses using this alternative categorical variable replicated analyses with the continuous scales and can be found in online supplements: https://osf.io/n95rm/

2 Note that when analyzing each decision separately in logistic regressions, volatility significantly predicted retirement plans, having insurance, and using savings but did not predict having a budget. These supplemental analyses are available at https://osf.io/fv4pu/

3 We also assessed participants’ discounting rate. In instructions taken from previous work (Frederick et al., 2002; Bartels and Rips, 2010), participants read “Most people don’t like to wait for their money but are willing to do so if the wait means they receive a little more money. This is called the point of indifference: The point of indifference is the point at which you think the higher amount is worth waiting for the specified amount of time. For how much money would you wait 1 year rather than taking $100 now?” and entered a value in the sentence “I would be indifferent between $100 tomorrow and $_____ in 1 year.” However, participants in this sample answered in a way that suggests they did not understand the question, with 9.6% entering a value less than $100 and 8% entering a value of more than $1000. In analyses, we log-transformed values to account for extreme negative skew, but results should be interpreted with caution. Greater discounting was linked with fewer planning decisions, *r*(973) = −0.11, *p* = 0.001. In a multiple regression, volatility was negatively linked to discounting, *B* = −0.04, *SE* = 0.01, *beta* = −0.14, *t*(959) = −4.14, *p* < 0.001. However, further analyses showed that this link was entirely driven by those participants exhibiting ‘reverse’ discounting (i.e., who entered values below 100), and volatility was not linked to discounting when these participants were excluded, *B* = −0.001, *SE* = 0.01, β = −0.003, *t*(888) = −0.10, *p* = 0.924.

4 Note that an alternative way to test the indirect effect in multiple regressions (using PROCESS v3.4, Hayes, 2017; Model 4, 5000 bootstrap samples) where volatility was entered as predictor, LOC and economic LOC as mediators, the financial planning index as dependent variable, and age, gender, education and income amount were entered as covariates confirmed significant indirect effects via LOC: B = −0.012, 95%CI[−0.03; −0.01], and via economic LOC: B = −0.011, 95%CI[−0.03; −0.01].

5 https://osf.io/29yvj/

6 We had also assessed participants’ discounting rate, using the same measure as in Study 1 (… “I would be indifferent between $100 tomorrow and $_____ in 1 year”). None of the participants reported values below $100, but many expressed confusion about this item to the experimenter and some did not answer this question, casting doubt on the validity of this measure. We log-transformed values to account for extreme negative skew. A logistic regression with log discounting and condition (volatile vs. stable) as predictors showed that greater discounting was significantly linked to the saving decision, *B* = −0.80, *SE* = 0.38, *Exp*(*B*) = 0.45, *p* = 0.034. Thus, greater discounting of the value of money in the future led to less likelihood to choose a delayed, but higher payout. However, given the extreme skew of the discounting measure and the verbally expressed confusion during the experiment, this measure should be interpreted with caution.
